# Present and Future Perspectives on Cell Sheet-Based Myocardial Regeneration Therapy

**DOI:** 10.1155/2013/583912

**Published:** 2013-12-04

**Authors:** Yoshiki Sawa, Shigeru Miyagawa

**Affiliations:** Department of Cardiovascular Surgery, Osaka University Graduate School of Medicine E1, 2-2 Yamadaoka, Suita, Osaka 565-0871, Japan

## Abstract

Heart failure is a life-threatening disorder worldwide and many papers reported about myocardial regeneration through surgical method induced by LVAD, cellular cardiomyoplasty (cell injection), tissue cardiomyoplasty (bioengineered cardiac graft implantation), *in situ* engineering (scaffold implantation), and LV restrictive devices. Some of these innovated technologies have been introduced to clinical settings. Especially, cell sheet technology has been developed and has already been introduced to clinical situation. As the first step in development of cell sheet, neonatal cardiomyocyte sheets were established and these sheets showed electrical and histological homogeneous heart-like tissue with contractile ability *in vitro* and worked as functional heart muscle which has electrical communication with recipient myocardium in small animal heart failure model. Next, as a preclinical study, noncontractile myoblast sheets have been established and these sheets have proved to secrete multiple cytokines such as HGF or VEGF *in vitro* study. Moreover, *in vivo* studies using large and small animal heart failure model have been done and myoblast sheets could improve diastolic and systolic performance by cytokine paracrine effect such as angiogenesis, antifibrosis, and stem cell migration. Recently evidenced by these preclinical results, clinical trials using autologous myoblast sheets have been started in ICM and DCM patients and some patients showed LV reverse remodelling, improved symptoms, and exercise tolerance. Recent works demonstrated that iPS cell-derived cardiomyocyte sheet were developed and showed electrical and microstructural homogeneity of heart tissue *in vitro*, leading to the establishment of proof of concept in small and large animal heart failure model.

## 1. Introduction

Therapeutic treatments using cells or cell-based tissues have been developed to regenerate the damaged myocardium associated with ischemic heart disease. This technique has already been evaluated in the clinical setting, using myoblasts [1] or bone marrow mononuclear cells (BM-MNCs) [2]. Although these studies demonstrated the feasibility and safety of this approach, the efficacy associated with this technology was generally insufficient to repair severe myocardial damage. Thus, a second generation of myocardial regenerative therapy, tissue-engineered cardiomyoplasty, is currently being developed. A large number of achievements concerning basic, preclinical, and clinical works about cell sheet technology have been done and this review summarizes recent advances in myocardial regeneration emerging from the development of cell sheet technology.

## 2. Development of Cell Sheet Technology

Cell-sheet techniques have been applied to several diseased organs, such as the heart [3], eye [4], and kidney [5], in the laboratory and the clinic. Cell sheets can be prepared on special dishes that are coated with a temperature-responsive polymer, poly(N-isopropylacrylamide) (PIPAAm), that changes from being hydrophobic to hydrophilic when the temperature is lowered. This change allows cells to be removed without EDTA or enzymatic treatment and without destroying the cell-cell or cell-extracellular matrix (ECM) interactions within the cell sheet.

Shimizu et al. used such temperature-sensitive culture dishes to develop a contractile chick cardiomyocyte sheet that exhibited a recognizable heart tissue-like structure and showed electrical pulsatile amplitude [6]. Next, they layered single-cell sheets to generate bilayer-cell sheets, forming an electrically communicative three-dimensional cardiac construct, which exhibited spontaneous and synchronous pulsation with electrical communication between the cell sheets, mediated by connexin 43. Furthermore, the cell sheets adhered together rapidly, as indicated by the presence of desmosomes and intercalated disks between them [7]. When the pulsatile cardiac tissue was implanted subcutaneously, it was found to assume a heart tissue-like structure and exhibited neovascularization and spontaneous beating for up to one year. The size, conduction velocity, and contractile force of the engrafted sheets increased in proportion to the host growth [8, 9].

Miyagawa et al. demonstrated that a neonatal cardiomyocyte sheet could communicate electrically with the host myocardium, as indicated by the presence of connexin 43, and changes in the QRS wave and action potential amplitude, leading to improved cardiac performance in a rat model of ischemic heart disease [3]. This study clearly showed electrical and morphological coupling between the cell sheet and host myocardium and that the cell sheet could contract synchronously with the beating of the host heart and improve the regional systolic function.

A detailed analysis of the vascularization process following cell sheet implantation was undertaken by Sekiya et al. These authors reported that the cardiomyocyte sheet expresses angiogenesis-associated genes and forms an endothelial cell network. Evidence was also presented suggesting that the vessels arising in the engrafted sheet migrate to connect with the host vasculature [10].

Myocardial tissue grafts engineered with cell sheet technology represent a promising therapy for repairing the damaged myocardium, but there may be some inherent limitations. For example, cellular treatment for heart failure may be not suitable for emergency situations. Another issue is that wide therapeutic use will require improvement in the uniformity in the quality of the cultured cells.

Recently, new medications that imitate the paracrine effects of cytokines in cell sheets have been reported, and the addition of such medications could improve the regenerative treatment for heart failure. It was reported that the direct introduction of a prostacyclin agonist into the damaged myocardium induced significant functional recovery in a canine model of dilated cardiomyopathy, via the upregulation of multiple cytokines, including HGF, VEGF, and SDF-1 [11]. Similarly, the implantation of an atelocollagen sheet containing a prostacyclin analogue induced improved cardiac function and a prolonged survival rate in a mouse model of acute myocardial infarction, accompanied by an enhanced expression of SDF-1 [12]. Recent work has also revealed that prostacyclin may be upregulated in the implanted myoblast sheet in the early phases after implantation in response to ischemic conditions and may in turn stimulate endothelial or smooth muscle cells to secrete multiple cytokines including HGF, VEGF, and SDF-1 (data not shown).

## 3. Experimental and Clinical Work on Myoblast Sheets

In the clinical setting, cellular cardiomyoplasty is reported to have potential regenerative capability, and a method using skeletal myoblasts has been evaluated in clinical trials and found to be relatively feasible and safe [13]. For tissue cardiomyoplasty, skeletal myoblasts are the cell source closest to being ready for clinical application at this time. Memon et al. demonstrated that the nonligature implantation of a skeletal myoblast sheet into a rat cardiac ligation model regenerated the damaged myocardium and improved global cardiac function, by attenuating cardiac remodeling via hematopoietic stem-cell recruitment and growth-factor release, with better restoration of the implanted cells than that obtained using needle injection [14]. In another study, the application of a skeletal myoblast sheet into a 27-week dilated cardiomyopathy hamster model resulted in the attenuated deterioration of cardiac performance accompanied by the preservation of alpha-sarcoglycan and beta-sarcoglycan expression in the host myocytes, and an inhibition of fibrosis, leading to prolonged survival rates [15]. In addition, the grafting of skeletal myoblast sheets attenuated cardiac remodeling and improved cardiac performance in a pacing-induced canine heart failure model [16]. Studies from our group have shown that myoblast sheets may improve cardiac performance via cytokines such as HGF or VEGF (XX).

The mechanism of recovery in the damaged myocardium has not been completely elucidated and may be very complicated. As mentioned above, cytokine release and hematopoietic stem-cell recruitment are possible mechanisms of regeneration; however, other regenerative mechanisms are likely to be involved as well. Skeletal myoblasts cannot beat synchronously with the host myocardium *in vitro* [17] or *in vivo* [18], and, thus, they do not appear to be functionally integrated. However, data from our human and porcine studies suggested that after myoblast sheet implantation, the diastolic dysfunction in the distressed region of the myocardium was significantly recovered compared with controls, leading to improved systolic function in the same region, without contraction of the implanted myoblasts (data not shown). Massive angiogenesis in the implanted region was detected histologically and appeared to be a critical feature associated with the improvement. Thus, we speculate that angiogenesis and the recovery of diastolic function are both major components of the regenerative mechanism in myoblast sheet implantation [19].

On the other hand, immunohistochemical analysis has indicated that the myoblast sheet may only survive for a few months after implantation. We speculate that in the early phases after implantation of the myoblast sheet, the ischemic conditions induce the upregulated expression of several cytokines by the myoblasts that promote their own survival. These cytokines then in turn enhance angiogenesis and the recruitment of stem cells, leading to improved blood perfusion to reactivate the damaged myocardium. The system may continue to be effective in spite of the short-lived myoblast sheet, due to long-term maintenance of the newly developed vasculature.

We recently initiated a clinical evaluation of autologous myoblast sheet implantation. We tested the technology in four patients who were using left ventricular assist devises (LVADs); three of the four patients showed functional recovery, and in two of the patients, the treatment provided a bridge to recovery [20]. Six years later, these two patients have no symptoms of heart failure. We have also implanted myoblast sheets into eight patients with ischemic cardiomyopathy and seven with dilated cardiomyopathy (who were not using LVADs). In that study, some of the patients exhibited left ventricle reverse remodeling and improvements in exercise tolerance and symptoms, with no major adverse cardiac events (MACEs) (data not shown). This clinical research program is ongoing, as we continue to evaluate patients with dilated cardiomyopathy and ischemic cardiomyopathy with and without the use of LVADs.

## 4. Other Types of Cell Sheets

In addition to cardiomyocytes and myoblasts, other types of cell sheets have been used effectively to improve cardiac performance. The transplantation of a mesenchymal stem cell (MSC) sheet onto the infarcted myocardium of rats resulted in increased anterior wall thickness and new vessel formation, accompanied by a low incidence of differentiation of the implanted cells to cardiomyocytes [21]. While the small number of differentiated cardiomyocytes may not have contributed to the observed improvement in systolic function in this study, the cell sheet exhibited self-propagating properties that promoted the generation of a thick-layered sheet. Although the MSC sheet exhibited a maximum thickness of approximately 600 *μ*m, which would not be strong enough to correct human end-stage heart failure [22], this method of self-propagation is a potential strategy for creating a thick-layered sheet *in vivo*, with the potential for cardiac tissue regeneration.

A further development in cell sheet technology is the creation of a cell sheet composed of two types of cocultured cells; this type of cell sheet was developed to enhance angiogenesis [23, 24]. The cocultured cell sheet, which combined fibroblasts and endothelial progenitor cells, enhanced blood vessel formation and led to functional improvement in a rat myocardial infarction model [24]. Cocultured cell sheets combining fibroblasts and human smooth muscle cells were found to accelerate the secretion of angiogenic factors *in vitro* and to increase blood perfusion *in vivo* by the formation of new vessels [25]. This enhanced effectiveness attained by coculturing two cell types is supported by another study in which the coimplantation of BMCs and myoblasts showed improved results compared to the transplantation of a single cell type in a canine model of ischemic cardiomyopathy [26].

Cell sheets composed of stem cell antigen-1- (sca-1-) positive, or kit-positive cells may represent additional promising approaches. Matsuura et al. demonstrated that sca-1-positive cell sheets could differentiate into cardiomyocytes *in vivo* and produce VCAM-1, leading to improved cardiac performance in a mouse model of myocardial infarction [27]. The administration of c-kit-positive stem cells has shown efficacy in animal models of cardiac dysfunction, and this approach is currently being tested in clinical trials in combination with coronary artery bypass grafting, with encouraging preliminary results [28]. In another study, a c-kit-positive cell sheet combined with endothelial progenitor cell injection was found to induce better functional recovery of endocardial scar tissue than that induced by the cell sheet alone, despite the poor transdifferentiation ability of the c-kit-positive cells into cardiomyocytes [29].

Many of the cell sources mentioned above demonstrate regenerative ability based on the paracrine effect of secreted cytokines; however, newly differentiated cardiomyocytes may be the best candidate cells to regenerate the damaged myocardium. In 2006, Takahashi and Yamanaka reported the development of induced pluripotent stem (iPS) cells that can differentiate into various types of cells, such as cardiomyocytes, cartilage, and nerve cells [30]. Since then, there have been many reports showing that cardiomyocytes derived from iPS cells demonstrate electrophysiological, functional, and microstructural similarities to native cardiomyocytes [31]. Cardiomyocyte sheets derived from human or mouse iPS cells that contract synchronously *in vitro* have been developed, and studies indicate that these cardiomyocyte sheets can contract *in vivo* as analyzed by X-ray diffraction with synchrotron radiation. The transplantation of these sheets leads to functional recovery with upregulated electrical potential in the scarred areas in large [32] and small animal myocardial infarction models [33].

Although preclinical studies appear promising, the safety of these artificially generated cells must be evaluated thoroughly before they can be used in the clinic. In addition, a potential limitation of iPS cell-derived cardiomyocytes may be the loss of cardiomyocytes due to ischemia after implantation. Recent studies have proposed supplemental strategies to avoid ischemia. In one study, the combination of an iPS-derived cardiomyocyte sheet with omentum, which has a rich vasculature network, resulted in retention of the implanted cardiomyocytes and enhanced functional recovery compared with the cardiomyocyte sheet alone [34]. In another study, the transplantation of a cardiomyocyte sheet containing iPS cell-derived endothelial cells led to enhanced functional recovery in a rat myocardial infarction model and increased survival of the implanted cardiomyocytes [35]. Thus, to successfully treat the severely damaged myocardium using iPS cell-derived cardiomyocyte sheets, additional strategies to increase angiogenesis and reduce ischemia may be required.

## 5. Advantages of Cell Sheet Technology

Studies on the original myoblast cell therapy, in which cells were directly injected into the myocardium, indicated that the proportion of injected cells surviving to engraft the infarcted myocardium was too low to be effective. This low level of engraftment may have been caused by the injected cells leaking out of the injected region and being carried to other organs, or due to mechanical stress resulting in cellular loss of function. The resulting rapid cell loss [14] limited the usefulness of the original myoblast cell therapy.

To overcome the problems associated with the intramyocardial injection of cells, many investigators have combined cell transplantation with protein or gene therapy [36], or with tissue-engineered techniques [3]. We have also developed a new cell delivery system that uses tissue-engineered myoblast grafts grown as cell sheets and have utilized animal studies to guide clinical trials. These studies showed that the viability of the transplanted cells was higher than that of injected cells, and that the transplanted myoblasts survived for at least 3 months in the cardiac tissue of a porcine model of heart failure treated with autologous myoblast sheets. Using tissue-engineered temperature responsive techniques, we found that the implanted cells could be applied in larger numbers, were viable during transplantation, and were not lost from the applied region. Furthermore, we showed that cell sheets could be engrafted onto the failed myocardium and contribute to the attenuation of cardiac dysfunction and remodeling [14].

In cell therapy for cardiac disease, life-threatening adverse events involving arrhythmogenicity are a potential risk in both animal models and human clinical trials [37]; however, life-threatening arrhythmias have not been observed during the clinical course of patients who have received autologous cell sheet transplants. In any case, arrhythmias can occur during the natural clinical course of severe heart failure, so their cause may not be easily determined. Procedures using needle injection may cause scars in the myocardium that could in turn induce arrhythmias. Our cell delivery techniques using cell sheets prepared on temperature-responsive culture dishes may carry less risk for the induction of arrhythmias. Myoblasts have a weak electrical potential, and it may be possible for these cells to induce arrhythmia if they survive in the myocardium. However, cell sheets may not be able to induce arrhythmia, since they are attached to the epicardium.

Another potential problem is the limited blood perfusion to the implanted cell sheets. Although the survival of implanted cells using the cell sheet technique has already been shown to exceed the cell survival using other delivery routes, the survival rate was still found to be relatively low when the cells were implanted on the epicardium with this technique [38]. Although we have reported that improved cardiac performance depends on the dose of implanted myoblast sheets, the use of too many cell sheets results in a reduced blood supply. Thus, additional strategies, such as combining myoblasts with angiogenic factors [36] or other types of cells [23] to establish a vasculature network, may be needed to solve this problem. One strategy discussed above, is the combination of a myoblast sheet with omentum tissue that has a rich vasculature network. One report recently demonstrated the effectiveness of this approach for retention of the implanted cell sheets [39]. This report also suggested that the implanted myoblast sheet might induce vasculature connections between arteries of the transplanted omentum and the native coronary arteries, suggesting the possibility of biocoronary artery bypass grafting. This method may also be used in conjunction with iPS cell-derived cardiomyocytes to generate an artificial thick cardiac structure with increased vascular connections.

## 6. Conclusions

In this review, we surveyed many exciting topics in the area of cell sheet technology for cardiac repair. Owing to these studies, some techniques have already been tested in clinical applications, but the mechanisms by which they improve cardiac function are only partially understood, and much of the technology is still in the early stages of development, both experimentally and in the clinic. Nevertheless, the field of clinical myocardial regenerative therapy holds much promise, and we expect to witness more progress in this innovative technology in the near future.
